# Effects of D‐Chiral Inositol on Amino Acid Metabolism in a PCOS Rat Model

**DOI:** 10.1155/ije/5563183

**Published:** 2026-06-12

**Authors:** Ting Zhao, Jinyang Zhang, XiaoXiao, JingZhu, Jiaqi Wu, Yanmeng Wei, Huacong Shi, Xiaomei Wu, Tao Yuan

**Affiliations:** ^1^ Department of Gynaecology, The First People’s Hospital of Yunnan Province, Kunming, Yunnan, China, ypfph.com; ^2^ The Affiliated Hospital of Kunming University of Science and Technology, Kunming, Yunnan, China, kmust.edu.cn; ^3^ Yunnan Province Clinical Research Center for Gynecological and Obstetric Disease, First People’s Hospital of Yunnan Province, Kunming, Yunnan, China; ^4^ Research Center of Molecular Medicine of Yunnan Province, Faculty of Life Science and Technology, Kunming University of Science and Technology, Kunming, Yunnan, China, kmust.edu.cn

**Keywords:** D-Chiral inositol, metabolome, polycystic ovary syndrome, transcriptome

## Abstract

**Background:**

Polycystic ovary syndrome (PCOS) is a common endocrine disorder that affects fertility through oligo weathering, hyperandrogenism, and polycystic morphology of the ovary. Currently, D‐chiral inositol (DCI) treatment of PCOS has yielded satisfactory results by targeting steroid biosynthesis, but a deeper understanding of the potential mechanisms and efficacy of this treatment regimen is needed. The aim of this study was to determine the effects of DCI use on endocrine and metabolic abnormalities in women with PCOS.

**Materials and Methods:**

A PCOS rat model was developed via letrozole induction. After successful modeling, letrozole was administered orally to the rats for 4 weeks (PCOS + DCI). Enzyme‐linked immunosorbent assay was used to examine changes in serum hormone levels in PCOS rats and PCOS + DCI rats, and transcriptomics was used to detect changes in the expression of related target genes and signaling pathways. Furthermore, amino acid–targeted metabolomics was performed.

**Result:**

DCI intervention effectively improved the symptoms of PCOS in the rats, including polycystic ovary‐like changes and serum sex hormone and insulin levels. Further transcriptomic validation showed significant changes in the expression of genes such as *Lamc2*, *Inhba*, and *Bdkrb2* in DCI‐treated rats. Metabolomic validation targeting amino end groups revealed that DCI effectively improved amino acid metabolism in PCOS rats.

**Conclusion:**

This study confirmed that DCI can not only alleviate the reproductive and metabolic abnormalities of PCOS phenotypically but also regulate gene expression and metabolic networks at the molecular level. Its therapeutic effect has the characteristics of being multitarget and systematic.

## 1. Introduction

Polycystic ovary syndrome (PCOS) is a complex condition associated with metabolic disorders, hormonal disorders, ovarian dysfunction, and menstrual irregularities and is the main cause of infertility in women of childbearing age [1, 2]. Current treatment strategies for PCOS include first‐line therapies such as lifestyle modifications and oral contraceptive use, along with targeted treatments for patients with ovulation disorders [3]^.^ The prevalence of metabolic syndrome in patients with PCOS is > 50%. The use of medications to reduce insulin levels can improve complications associated with hyperinsulinemia and hyperandrogenism; thus, it has become a new approach to treat PCOS [4]. Given this emerging focus on insulin sensitization therapies, naturally occurring compounds such as D‐Chiral inositol (DCI) have garnered attention for their potential in managing PCOS‐related metabolic and reproductive abnormalities.

DCI is a chiral hydroxycyclohexanol primarily found in plants such as buckwheat and soybeans [5]. DCI has unique physiological functions, including promoting liver lipid metabolism; enhancing insulin sensitization; reducing blood sugar levels; treating PCOS; regulating hormone levels; and exerting antioxidant, antiaging, and anti‐inflammatory effects. Furthermore, it can improve the effects of PCOS on metabolism and the reproductive system [6, 7]. A preliminary study has reported that DCI reduces the blood glucose level in rats with Type 2 diabetes by regulating the gene and protein expression of PI3K and Akt [8]. Chiral inositol improves the energy status and quality of oocytes. Moreover, DCI can rapidly reduce peripheral hyperinsulinemia; thus, it is beneficial for treating PCOS [9]. Most previous studies have focused on the ability of chiral inositol to improve ovulation rate and fertility in women with PCOS; however, the underlying mechanism has not been studied.

It is worth noting that the metabolic disorders in PCOS extend beyond sugar and lipid metabolism. In recent years, the imbalance in amino acid metabolism has gradually gained attention as one of the potential pathological features of PCOS. Clinical studies have found that in PCOS patients, especially those with insulin resistance, the levels of branched‐chain amino acids (such as leucine, isoleucine, and valine) and aromatic amino acids in the circulation are significantly elevated. This metabolic characteristic is closely related to the degree of insulin resistance [10, 11]. Therefore, exploring the amino acid metabolic characteristics under PCOS conditions is of great significance for revealing its complex pathological mechanism. Although DCI has been proven to improve the metabolic and reproductive parameters of PCOS, current studies mostly focus on its role in regulating sugar and lipid metabolism. There is currently a lack of relevant data on whether DCI can correct the amino acid metabolic disorders related to PCOS and its specific mechanism [7, 12].

Currently, there is no reliable treatment for PCOS. In recent years, clinical treatment methods for PCOS have gradually improved, with a trend toward balancing prevention and standard treatment [13, 14]. Regardless of the diagnostic criteria used for PCOS, this syndrome is known to be related to metabolism, hormones, and reproduction and requires appropriate treatment. Thus, the benefits of using DCI in PCOS treatment are apparent. In this study, a rat model of PCOS was used to determine the effects of DCI on serum hormone levels, such as luteinizing hormone (LH), follicle‐stimulating hormone (FSH), estradiol (E2), and testosterone (T), which are associated with PCOS symptoms, in the blood and ovarian tissues. To the best of our knowledge, this is the first study to explore the effects of DCI on amino acid metabolism in rats and investigate the signaling pathways involved in these effects.

## 2. Methods

### 2.1. PCOS Rat Model Construction and Animal Treatment

Thirty healthy and clean‐grade female SD rats, with an average weight of 200 g, were selected and acclimated for 3 days. All animals were fasted for 6–8 h before the experiment. The model group (20 rats) was given letrozole at a dose of 1 mg/kg/d. The letrozole suspension was directly injected into the rats’ stomachs through the gastric tube at a fixed time every day. The healthy control group (*n* = 10) was given normal saline by gavage, and this was performed for 28 consecutive days. Subsequently, the model was verified by observing the estrous cycle, serum hormone levels, and ovarian tissue morphology. After successful modeling, the intervention was carried out. The normal group (*n* = 10) was given 2 mL of normal saline by gavage for 4 consecutive weeks; the PCOS group (*n* = 10) was given 2 mL of normal saline by gavage for 4 consecutive weeks; the PCOS + DCI group (*n* = 10) was given DCI solution (50 mg/kg/d) by gavage for 4 consecutive weeks [6].

### 2.2. Detection of Estrous Cycle in PCOS Rats

Every morning, rats were captured, and normal saline was extracted and injected into the vagina of female rats. The rats were rinsed three to five times. Then, the following process was followed: Take 10 µL of the prepared turbid physiological saline and use another pipette to draw the crystal violet staining solution for staining. Fix the dyeing solution with a cover plate, mix thoroughly, and then absorb the excess liquid with absorbent paper. Then, place it under an optical microscope for observation. Vaginal smears are used to monitor the estrous cycle, which is divided into four stages: pre (P), estrous (E), next estrous (M), and estrous (D). Estrous cycle disorder is defined as a significantly prolonged cycle (≥ 6 days) or no change in a certain stage for ≥ 3 days.

### 2.3. Serum Hormone Level Detection

The rats were divided into the model group, the modeling group, and the DCI intervention group (*n* = 3 for each group). Blood samples were collected from each group of rats at 10 a.m. Using the enzyme‐linked immunosorbent assay (ELISA) kit for rats (Beyotime Biotechnology Co., Ltd., China), according to the manufacturer’s instructions, the serum levels of FSH, LH, estradiol (E2), testosterone (T), and anti‐Müllerian hormone (AMH) were detected. The absorbance was measured at 450 nm.

### 2.4. RNA Extraction and Library Construction

Total RNA was isolated and purified using TRIzol reagent (Invitrogen, Carlsbad, CA, USA) according to the manufacturer’s instructions. A NanoDrop ND‐1000 spectrophotometer (NanoDrop, Wilmington, DE, USA) was used to determine the concentration and purity of isolated RNA in each sample. RNA integrity was assessed using the Agilent Bioanalyzer 2100 (Agilent, CA, USA) with an RIN value of > 7.0 and further confirmed using denatured agar‐gel electrophoresis. Poly(A) RNA was purified from 1 μg of total RNA using Dynabeads Oligo (dT) 25‐61005 (Thermo Fisher, CA, USA). The Magnesium RNA Fragmentation Module (cat. no. e6150) was then used to fragment poly (A) RNA. The RNA fragments were reverse transcribed to cDNA using SuperScript II Reverse Transcriptase (cat. no. 1896649; Invitrogen). The cDNA was used to synthesize U‐labeled second‐stranded DNA with *Escherichia coli* DNA Polymerase I (cat. no. m0209; NEB), RNase H (cat. no. m0297; NEB), and dUTP solutions (cat. no. R0133; George Fisher). Bases were then added to the blunt ends of each strand for ligation to the index adapter. Each adapter contained a T‐base overhang to facilitate ligation to the A‐tail fragment of DNA. A single‐ or dual‐index adapter was ligated to the fragment, and then the AMPure XP beads were used for sized selection (cat. no. m02087; Inside). The U‐labeled second‐stranded DNA was treated with a heat‐labile UDG enzyme. The average insert size of the final cDNA library was 300 ± 50 bp. Finally, we performed 2 × 150‐bp paired end sequencing (PE150) on Illumina NovaSeq 6000 (LC‐Bio Technology Co., Ltd., Hangzhou, China) following the manufacturer’s recommended protocol.

### 2.5. Bioinformatic Analysis of RNA Sequencing Data

FASTQ (https://github.com/OpenGene/fastp) was used to trim the adapters, remove low‐quality bases, and correct wrong bases, using the default parameters. fastp was used to verify the sequence quality. We used HISAT2 (https://ccb.jhu.edu/software/hisat2) to map these reads against *Homo sapiens* GRCh38 reference genome. StringTie (https://ccb.jhu.edu/software/stringtie) was used for RNA assembly with default parameters. GFFCompare (https://github.com/gpertea/gffcompare/) was used to merge the transcriptomes of all samples into a comprehensive transcriptome. After generating the final transcriptome, StringTie was used to estimate the expression levels of all transcripts by calculating FPKM (FPKM = [total exon_ fragments/[mapped reads {millions} × exon length {kB}]]). The differentially expressed mRNAs with fold change > 2 or fold change < 0.5 and with parametric *F*‐test comparing nested linear models (*p* < 0.05) were identified using the R package edgeR (https://bioconductor.org/packages/release/bioc/html/edgeR.html).

### 2.6. Amino Acid Metabolomics

#### 2.6.1. Reagents and Instruments

An ultra‐high‐performance liquid chromatography system coupled to a tandem mass spectrometry (UHPLC‐MS/MS) system (ExionLC AD UHPLC‐QTRAP 6500+; AB SCIEX Corp., Boston, MA, USA) was used.

#### 2.6.2. Metabolite Extraction

The diluted samples were vortexed and added to distilled water. Thereafter, 50 μL of the sample was homogenized with 200 μL of a 1:1 acetonitrile/methanol mixture containing internal standards, followed by thorough vortexing. The sample was then placed on ice for 30 min and centrifuged at 12,000 rpm for 10 min. Finally, the supernatants were collected for subsequent analysis using an LC‐MS/MS system.

#### 2.6.3. HPLC‐MS/MS Analysis

LC‐MS/MS analysis was performed using the ExionLC AD system (SCIEX) coupled with the QTRAP6500+ mass spectrometer (SCIEX; Novo Gold Co., Ltd., Beijing, China). Chromatography was performed on the XSelect HSS T3 (2.1 × 150 mm, 2.5 μm) column at a flow rate of 0.4 mL/min with positive/negative ion mode and gradient elution for 20 min. We used 0.1% formic acid–water as Eluent A and 0.1% formic acid–acetonitrile as Eluent B [15]. The solvent gradient was as follows: 2% B, 2 min; 2%–100% B, 15.0 min; 100% B, 17.0 min; 100%–2% B, 17.1 min. The QTRAP6500+ mass spectrometer was operated in the positive ion mode under the following conditions: curtain gas pressure, 35 psi; impact gas pressure, medium; IonSpray voltage, 5500 V; temperature, 550°C; ion source Gas 1 pressure, 60 psi; and ion source Gas 2 pressure, 60 psi. The QTRAP6500+ mass spectrometer was operated in the negative ion mode under the following conditions: curtain gas pressure, 35 psi; impact gas pressure, medium; IonSpray voltage, −4500 V; temperature, 550°C; ion source Gas 1 pressure, 60 psi; and ion source Gas 2 pressure, 60 psi [16, 17].

### 2.7. Statistical Analysis

The SPSS 21.0 software (IBM Corp., Armonk, NY, USA) was used for statistical analysis. The experimental data are expressed as mean ± standard deviation (SD) or mean ± standard error of the mean. One‐way analysis of variance was used for comparison between groups, and the least significant difference test was used for pairwise comparisons. Results with *p* < 0.05 were considered statistically significant.

## 3. Results

### 3.1. Hematoxylin–Eosin (HE) Staining Showed Changes in Ovarian Morphology After DCI Treatment

**FIGURE 1:**
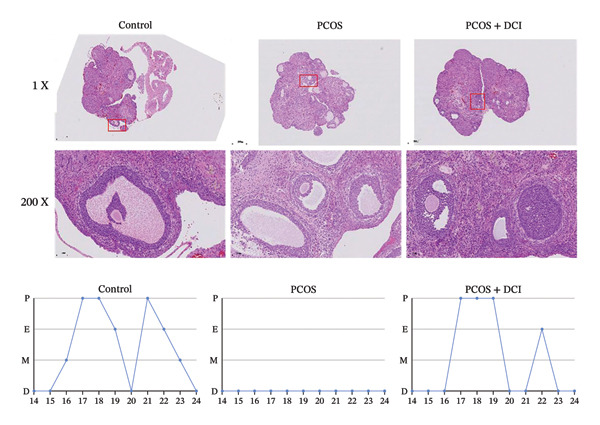
Hematoxylin–eosin staining analysis of ovarian morphological changes after D‐chiral inositol treatment and representative images of estrous cycles in different groups.

The HE staining results are shown in Figure 1. Cells in the control group were arranged neatly, whereas those in the PCOS group proliferated into the surrounding tissues and showed an increased number of vesicles and disordered cell arrangement. Compared to the model group, the PCOS + DCI group exhibited neatly arranged cells, reduced number of vesicles, and small vesicles. The presence of more than 12 immature small vesicles was considered polycystic. In addition, the normal estrous cycle of rats in the normal group (preestrous, estrous, postestrous, and interestrous periods) lasted approximately 4‐5 days. The estrous cycles of rats in the PCOS group and the PCOS + DCI group were disordered, or there was a prolonged stay at a certain period.

### 3.2. Changes in the Levels of Biochemical Indicators

**FIGURE 2:**
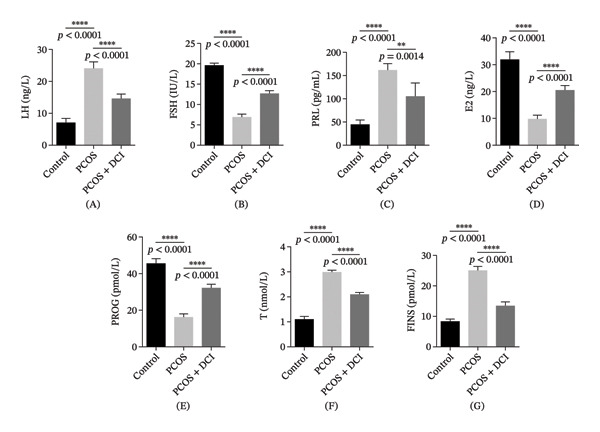
Changes in LH, FSH, PRL, E2, PROG, T, and FINS levels after DCI intervention. (A) Changes in LH expression after DCI intervention. (B) Changes in FSH expression after DCI intervention. (C) Changes in PRL expression after DCI intervention. (D) Changes in E2 expression after DCI intervention. (E) Changes in PROG expression after DCI intervention. (F) Changes in T expression after DCI intervention. (G) Changes in FINS expression after DCI intervention.

ELISA kits were used to determine the expression levels of LH, FSH, prolactin (PRL), estradiol (E2), progesterone (PROG), testosterone (T), and fasting insulin (FINS). Compared to those in the control group, the expression of FINS, LH, PRL, and T significantly increased, and that of FSH, ROG, and E2 significantly decreased in the PCOS group. Compared to the PCOS group, the PCOS + DCI group showed a significant decrease in FINS, LH, PRL, and T expression and a significant increase in FSH, PROG, and E2 expression (Figures 2A–G).

### 3.3. Transcriptomic Analysis of Differences in Gene Expression After DCI Treatment

**FIGURE 3:**
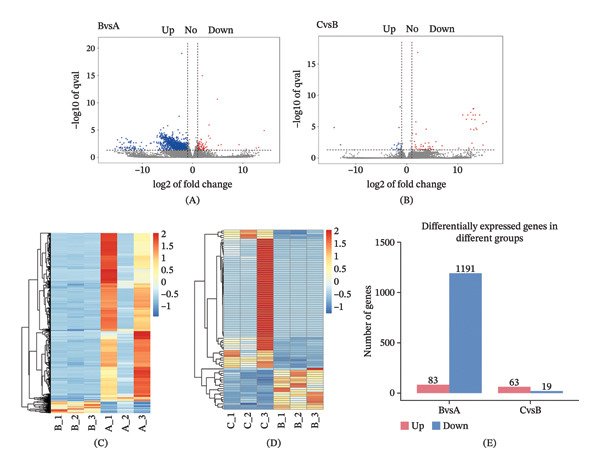
Differences in the serum transcriptomic profiles of rats with PCOS after DCI treatment. (A) Volcano plot showing the differences in the serum transcriptomic profiles between the PCOS model (B) and normal groups (A); (B) volcano plot showing the differences in the serum transcriptomic profiles between the DCI intervention (C) and PCOS model groups (B); (C) differential gene clustering diagram between the PCOS model (B) and normal groups (A); (D) differential gene clustering diagram between the DCI intervention (C) and PCOS model groups (B). Vertical clustering represents the clustering of samples, whereas horizontal clustering represents the clustering of metabolites. The shorter the clustering branch, the higher the similarity. Horizontal comparisons can indicate the relationship between clusters of metabolite content between groups. (E) Bar chart displaying the differences in transcriptome gene expression among different groups.

To visually observe the differential expression of genes and eliminate biological variations, we evaluated the differentially expressed genes between the PCOS model and normal groups, as well as between the DCI intervention and PCOS model groups, in terms of fold change and significance level. A volcano plot is shown in Figure 3A. The threshold for analysis in this experiment is set as log2 fold change ≥ 1, and we used the Benjamini–Hochberg (BH) method to correct the original *p* values to control the false discovery rate (FDR). According to the reviewers’ suggestions, we updated the significance threshold from the original *p* < 0.05 to *p*. adjust < 0.05 (FDR < 0.05). The results revealed 1274 significantly differentially expressed genes between the PCOS model group and the normal group: 83 upregulated genes and 1191 downregulated genes. In contrast, the DCI intervention group and PCOS model group exhibited 82 significantly differentially expressed genes, with 63 upregulated and 19 downregulated genes.

**FIGURE 4:**
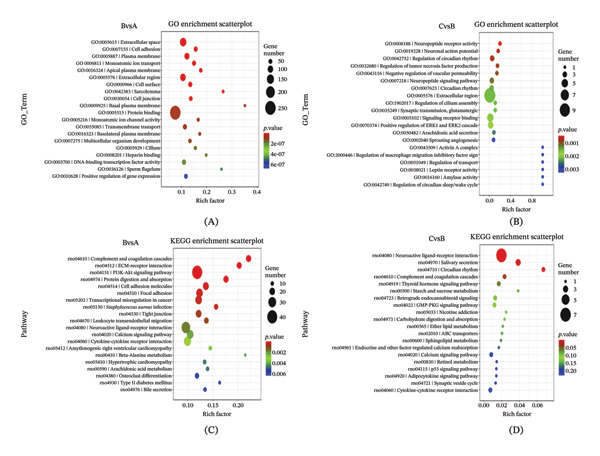
Z‐Scores and results of KEGG analysis of differentially expressed genes among the groups. (A and C) GO and KEGG enrichment scatter plots of the PCOS model (B) and normal groups (A). (B and D) GO and KEGG enrichment scatter plots of the DCI intervention (C) and PCOS model groups (B).

Kyoto Encyclopedia of Genes and Genomes (KEGG) pathway enrichment analysis of differentially expressed genes was performed using a homologous annotation system, and the top 20 pathways with the highest significance were identified. The results showed that the differential genes in the PCOS model group (B) and the normal group (A) were enriched in a total of 286 signaling pathways. Among them, 50 pathways such as the PI3K/Akt signaling pathway, cAMP signaling pathway, ECM receptor, cytokine–cytokine receptor interaction, and cell adhesion molecules were significantly enriched (*p* < 0.05). The differentially expressed genes between the DCI intervention group and the PCOS model group were enriched in 65 signaling pathways, with four significant (*p* < 0.05) pathways, namely, neuroactive ligand–receptor interactions, saliva secretion, circadian rhythm, and complement and coagulation cascade pathways (Figures 4A–D).

### 3.4. Transcriptome Analysis Results Revealed Differential Gene Expression After DCI Treatment

**FIGURE 5:**
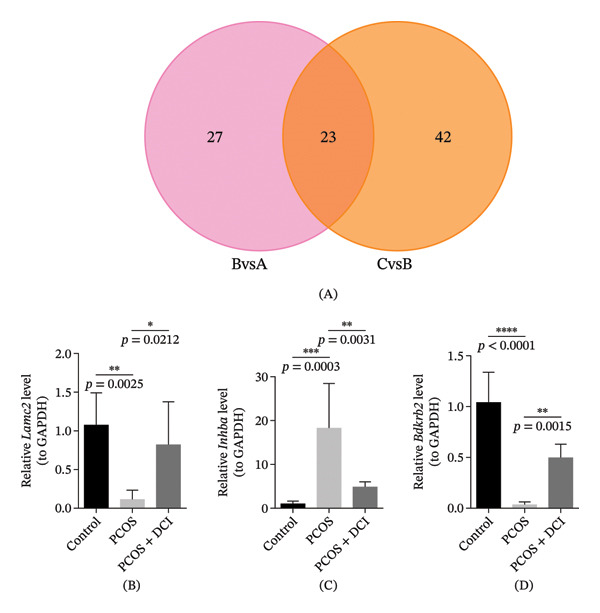
Transcriptome analysis results of differential gene expression after DCI treatment. (A) Gene expression trends in the normal (A), PCOS (B), and PCOS + DCI groups (C). (B–D) Differential expression of the target genes *Inhba*, *Lamc2*, and *Bdkrb2* in each group.

To screen for characteristic genes associated with the intervention conditions of the model, we intersected the significantly enriched KEGG pathways (*p* < 0.05) between two groups of differentially expressed genes. Specifically, we intersected all pathways enriched in the PCOS + DCI vs. PCOS comparison with those significantly enriched in the PCOS vs. control comparison. As a result, we identified 23 common pathways (Figure 5). The aim of this study was to investigate the effects of DCI intervention on PCOS. Given that DCI is an insulin metabolism signaling molecule, we selected nine signal transduction–related pathways (KEGG Level 2 classification) from the common pathways and extracted the differentially expressed genes enriched in these nine pathways. Based on the intervention conditions of the model and the trend of gene expression changes, we screened genes with upregulated and downregulated expression in both PCOS vs. control and PCOS + DCI vs. PCOS in the same pathway or genes that were downregulated in both PCOS vs. control and PCOS + DCI vs. PCOS in the same pathway. To screen the characteristic genes, based on the intervention treatment conditions of the model, we took the intersection of the pathways with significant KEGG enrichment (*p* < 0.05) of the two groups of differentially expressed genes. According to the intervention treatment conditions of the model and the changing trend of gene expression, we screened genes that were simultaneously differentially upregulated in BvsA and differentially downregulated in CvsB, or genes that were simultaneously differentially downregulated in BvsA and differentially upregulated in CvsB in the same pathway. A total of 3 genes met the conditions. Three genes that met these conditions, *Inhba*, *Lamc2*, and *Bdkrb2*, were further validated.

### 3.5. Comparison of Amino Acid Metabolite Profiles After DCI Intervention

**FIGURE 6:**
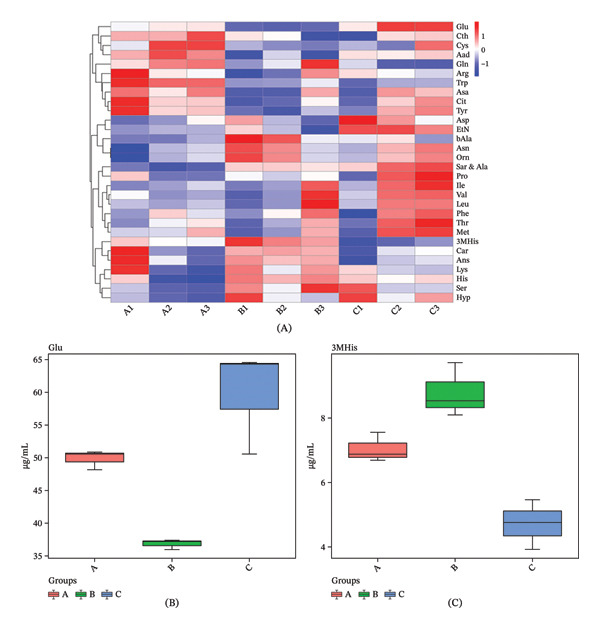
Comparison of lipid metabolism sequencing results after DCI treatment. (A) Heatmap of amino acid expression in the normal (A), PCOS (B), and PCOS + DCI (C) groups. (B) Box plots of two different amino acids in each group.

An important aspect of amino acid–targeted metabolomics is searching for characteristic amino acid metabolites, particularly screening of differentially expressed amino acids. Univariate statistical analysis was performed to identify statistically significant differential variables. This differential analysis did not focus on multiple differences and only screened differentially expressed metabolites with *p* < 0.05. The results revealed seven, six, and three differentially expressed metabolites in the PCOS vs. control, PCOS + DCI vs. control, and PCOS + DCI vs. PCOS comparisons, respectively. According to the model processing method, the amino acids whose expression changed following modeling and drug treatment were Glu and 3‐methylhistidine (3MHis). Glu expression decreased in the model group and increased after drug treatment, whereas 3MHis expression increased in the model group and decreased after drug treatment (Table 1 and Figure 6).

**TABLE 1:** Comparison of differentially expressed amino acids among the healthy control (A), PCOS (B), and PCOS + DCI groups (C).

Group A	Group B	Parameter	Fold change	*p* value
B	A	Ser	1.235294118	0.048199587
B	A	Sar and Ala	1.436986301	0.007554581
B	A	Glu	0.738810955	**0.00089381** ^∗^
B	A	3MHis	1.24704212	**0.047944165** ^∗^
B	A	Aad	0.322738386	0.022732036
B	A	Tyr	0.751344511	0.03283746
B	A	Trp	0.585464334	0.002597817
C	A	Gln	0.702203857	0.017964129
C	A	Sar and Ala	1.615068493	0.00818687
C	A	Hyp	1.263661202	0.044289216
C	A	3MHis	0.670137246	**0.017316588** ^∗^
C	A	EtN	1.394171779	0.002122313
C	A	Trp	0.530282638	0.001406418
C	B	Glu	1.622965642	0.037088017
C	B	His	0.926587302	0.036410332
C	B	3MHis	0.537381404	**0.003498617** ^∗^

^∗^ *p* < 0.05.

**FIGURE 7:**
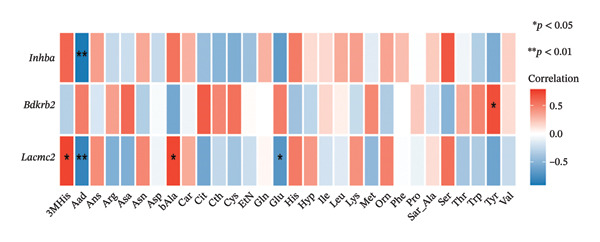
Heatmap analysis of the correlation between target genes and amino acids.

We conducted Pearson correlation analysis of the key genes *Lamc2*, *Inhba*, and *Bdkrb2* and amino acid metabolites to investigate the relationship between key genes and amino acid metabolism. The results showed a significant negative correlation between *Inhba* and aminoadipic acid (Aad). *Bdkrb2* significantly positively correlated with Tyr (amino acid). *Lamc2* significantly positively correlated with 3MHis and beta‐alanine and significantly negatively correlated with Aad and glutamic acid (Glu) (Figure 7).

## 4. Discussion

In this study, we first investigated the effects of DCI on the ovarian function, sex hormone levels, and serum metabolic levels of the rat model of PCOS. The results showed that after DCI intervention, the morphology of polycystic ovaries and the serum sex hormone levels were significantly improved (*p* < 0.05). In addition, we conducted transcriptome and metabolome analyses on the rat serum and found that the expressions of genes such as *Lamc2*, *Inhba*, and *Bdkrb2* had significant changes. Moreover, in the comparisons between the PCOS group and the control group, the PCOS plus DCI group and the control group, and the PCOS plus DCI group and the PCOS group, there were 7, 6, and 3 differentially expressed metabolites, respectively. Among them, Glu and 3MHis amino acids were different in all three groups (*p* < 0.05).

The research represented by Professor Genazzani’s team has provided core evidence for the clinical application of DCI. Randomized controlled trials have clearly shown that supplementing DCI can effectively improve insulin sensitivity, reduce androgen levels, and restore ovarian function in patients with PCOS [18]. Further research has found that the effectiveness of DCI treatment is not absolute; it fundamentally depends on the integrity of the homeostasis of inositol metabolism in the patient’s body. DCI is mainly transformed in the body from its isomer myo‐inositol through the action of inositol heteroisomerase. Therefore, the normal expression and function of this enzyme are the core physiological prerequisites for maintaining the level of DCI in the body and thereby ensuring the therapeutic effect of exogenous supplementation or endogenous transformation. It is worth noting that there are significant individual differences in this key premise. In people with a family history of Type 1 or Type 2 diabetes, there is a genetic predisposition that leads to a decrease in the expression or activity of ectoisomerase [19]. This study initially explores the clinical application potential of DCI in the treatment of PCOS, providing a basis for its future clinical promotion.

Since 2003, transvaginal ultrasound examination has been regarded as an important method for diagnosing PCOS, especially for determining the presence of 12 or more follicles in each ovary and the enlargement of the ovarian volume [20]. In this study, we observed that DCI therapy could effectively increase the number of mature follicles in rats with PCOS and significantly reduce the number of immature follicles. Chiral inositol and its stereoisomers are mediators of insulin action and are insulin sensitizers [21]. In this study, through DCI intervention, the free T level in PCOS rats induced by letrozole decreased, and the hormone levels, such as LH, FSH, and E2, changed. This finding indicates that the menstrual cycle and fertility have changed [22, 23]. In addition, the biosynthesis of high androgen levels in PCOS patients may originate from insulin resistance and the disorder of ovarian CI ratio. The increase in androgen levels in PCOS patients may be related to the decrease in DCI levels, thereby causing insulin resistance [7]. In our study, we observed that exogenous supplementation of DCI effectively increased the FINS level. These studies are consistent with the results of Sacchi S et al.′s research. The team discovered through the key model of in vitro culture of human granulosa cells that DCI specifically antagonizes the effect of insulin and can precisely inhibit the abnormal signal of insulin‐stimulating androgen synthesis locally in the ovary [24].

Metabolomics, a downstream supplement to transcriptomics, involves the universal analysis of physiological conditions in biological systems. It represents the final state of genetic regulation and its effects on intracellular enzyme activities and endogenous biochemical reactions. Women with PCOS are at a high risk of developing complications related to metabolic disorders [25, 26]. Previous studies have reported abnormalities in steroid hormone biosynthesis, lipid and carbohydrate metabolism, and amino acid metabolism in patients with PCOS [25]. In a previous study, compared with those in the control group, the plasma levels of glutamate, acetone, citrate, and histidine significantly reduced in patients with PCOS [27]. In our study, we observed that abnormalities in glutamate and histidine levels significantly improved in PCOS rats after DCI intervention. DCI can increase glucose uptake by cells and participate in FSH activity, which is crucial for glycogen synthesis and is involved in insulin‐induced ovarian androgen overproduction [28, 29]. Glutamate and histidine play important roles in the glucose metabolism pathway; the specific mechanisms and regulatory effects of DCI require further exploration.

In the KEGG pathway enrichment analysis, the differentially expressed genes between the PCOS model group and the normal group were significantly enriched in multiple pathways related to metabolic regulation and inflammatory responses. These pathways have been widely reported to be involved in the pathophysiological process of PCOS. Among them, the PI3K/Akt signaling pathway plays a key role in insulin resistance and ovarian dysfunction; it is notable that after DCI intervention, the pathways significantly enriched by the differentially expressed genes showed significant differences from the PCOS model group, and the significantly enriched pathways included neural activity ligand–receptor interaction, salivary secretion, and circadian rhythm pathways. These pathway changes may reflect the potential mechanism of DCI in improving metabolic disorders and inflammatory states related to PCOS. Although the research on these pathways in PCOS is not sufficient, this result provides new clues for further revealing the molecular mechanism of DCI intervention in PCOS.

This study also revealed the potential correlations between *Lamc2*, *Inhba*, *Bdkrb2*, and amino acid metabolites through correlation analysis. The significant association of *Lamc2* with 3MHis and Glu suggested that the extracellular matrix remodeling in PCOS might be coupled with enhanced myoprotein catabolism and the impairment of Glu‐mediated antioxidant defense system [30]. The decrease in Glu levels, as a precursor for glutathione synthesis, might directly exacerbate the oxidative stress in the ovarian local area of PCOS, thereby worsening insulin resistance [31, 32]. The increase in Glu levels after DCI intervention might be one of the mechanisms by which it improves oxidative stress. Similarly, the increase in 3MHis in the model group might reflect the abnormal activation of the mTOR signaling pathway under the PCOS state, leading to a high turnover rate of proteins. These amino acid metabolites may not only be disease markers but also serve as a bridge connecting gene expression differences with hormonal disorders and metabolic disorders [11, 33]. However, this study has not yet been able to directly confirm the regulatory effects of *Lamc2* or *Inhba* on specific amino acid transporters or metabolic enzymes through in vitro cell experiments or gene intervention methods. This will be the focus of the next research.

Although this study has confirmed the improvement effect of DCI on the PCOS rat model from multiple dimensions, including phenotype, gene transcription, and metabolic levels, there are still several limitations: First, the rat model used in this study to simulate PCOS was induced by letrozole. Although this model can well reproduce some reproductive and endocrine abnormalities, it cannot fully replicate the complex pathological and physiological characteristics of human PCOS patients. Second, as an exploratory study to initially investigate the intervention effect of DCI, this experiment only used a single effective concentration for observation and did not set up different dose gradients to evaluate its dose–response relationship. Further research should optimize the drug administration plan based on this. Moreover, the intervention period of 4 weeks is relatively short. There is a lack of in‐depth observation on the long‐term efficacy of DCI, the recurrence risk after discontinuation, and potential toxic side effects.

## 5. Conclusion

In this study, we observed that DCI treatment effectively improved the symptoms of PCOS in rats, including polycystic ovary–like changes and serum sex hormone and insulin levels. Further transcriptomic verification revealed significant regulatory changes in the expression of genes such as *Lamc2*, *Inhba*, and *Bdkrb2* in rats with PCOS after DCI treatment. In addition, amino end‐targeted metabolomic validation revealed that DCI effectively improved amino acid metabolism in PCOS rats.

## Author Contributions

Ting Zhao and Tao Yuan: conceptualization, methodology, and writing–original draft. Jinyang Zhang and Xiaomei Wu: formal analysis, data curation, and visualization. XiaoXiao, Jiaqi Wu, and JingZhu: investigation, resources, and writing–review and editing. Yanmeng Wei and Huacong Shi: supervision, project administration, and funding Acquisition.

## Funding

This work received financial support from the Xingdian Talent Support Program for Medical and Health Talents (XDYC‐YLWS‐2023‐0073), the Yunnan Provincial Center for Obstetrics and Gynecology Research (2026YJZX‐FC05 and 2026YJZX‐FC11), the Key Laboratory of Birth Defects and Genetic Diseases Research in Yunnan Province (2022ZDFKT002), the Central Government Guidance Fund for Local Science and Technology Development (202407AB110013), the Medical Reserve Talent of Yunnan Provincial Health Commission (H‐2024003), and the National Natural Science Foundation of China (82460299).

## Ethics Statement

This study was approved by the Experimental Animal Ethics Committee of Kunming University of Science and Technology (approval number: 82460299).

## Conflicts of Interest

The authors declare no conflicts of interest.

## Data Availability

Research data are not shared.

## References

[1] Yang J. and Chen C. , Hormonal Changes in PCOS, Journal of Endocrinology. (2024) 261, no. 1, 10.1530/joe-23-0342. 38285626

[2] Singh S. , Pal N. , Shubham S. et al., Polycystic Ovary Syndrome: Etiology, Current Management, and Future Therapeutics, Journal of Clinical Medicine. (2023) 12, no. 4, 10.3390/jcm12041454. PMC9964744 36835989

[3] Mimouni N. E. H. and Giacobini P. , Polycystic Ovary Syndrome (PCOS): Progress Towards a Better Understanding and Treatment of the Syndrome, Comptes Rendus Biologies. (2024) 347, no. G1, 19–25, 10.5802/crbiol.147. 38639155

[4] Mansour A. , Noori M. , Hakemi M. S. et al., Hyperandrogenism and Anthropometric Parameters in Women With Polycystic Ovary Syndrome, BMC Endocrine Disorders. (2024) 24, no. 1, 10.1186/s12902-024-01733-y. PMC11438141 39333998

[5] Laganà A. S. , Myers S. H. , Forte G. et al., Inositols in Treating Polycystic Ovary Syndrome and Non-Insulin Dependent Diabetes Mellitus: Now and the Future, Expert Opinion on Drug Metabolism & Toxicology. (2024) 20, no. 1-2, 61–72, 10.1080/17425255.2024.2306851. 38226638

[6] Yang H. , Lee S. R. , Jo S. L. et al., The Improvement Effect of D-Chiro-Inositol and *Ecklonia cava* K. in the Rat Model of Polycystic Ovarian Syndrome, Frontiers in Pharmacology. (2022) 13, 10.3389/fphar.2022.905191. PMC9343876 35928256

[7] Lete I. , Martínez A. , Lasaga I. , Centurión E. , and Vesga A. , Update on the Combination of Myo-Inositol/D-Chiro-Inositol for the Treatment of Polycystic Ovary Syndrome, Gynecological Endocrinology. (2024) 40, no. 1, 10.1080/09513590.2023.2301554. 38239032

[8] Cheng F. , Han L. , Xiao Y. et al., D-Chiro-Inositol Ameliorates High Fat Diet-Induced Hepatic Steatosis and Insulin Resistance via PKCε-PI3K/AKT Pathway, Journal of Agricultural and Food Chemistry. (2019) 67, no. 21, 5957–5967, 10.1021/acs.jafc.9b01253. 31066268

[9] Unfer V. and Porcaro G. , Updates on the Myo-Inositol Plus D-Chiro-Inositol Combined Therapy in Polycystic Ovary Syndrome, Expert Review of Clinical Pharmacology. (2014) 7, no. 5, 623–631, 10.1586/17512433.2014.925795. 24898153

[10] Jia F. C. and Li X. L. , Role of Branched-Chain Amino Acids in Metabolic Changes of Polycystic Ovary Syndrome, Obstetrical and Gynecological Survey. (2024) 79, no. 6, 343–347, 10.1097/ogx.0000000000001272. 38896430

[11] Mu L. , Ye Z. , Hu J. et al., PPM1K-Regulated Impaired Catabolism of Branched-Chain Amino Acids Orchestrates Polycystic Ovary Syndrome, EBioMedicine. (2023) 89, 10.1016/j.ebiom.2023.104492. PMC9986518 36863088

[12] Lentini G. , Querqui A. , Monti N. , and Bizzarri M. , PCOS and Inositols—Advances and Lessons We are Learning. A Narrative Review, Drug Design, Development and Therapy. (2025) 19, 4183–4199, 10.2147/dddt.s524718. 40420946 PMC12104671

[13] Banaszewska B. , Pawelczyk L. , and Spaczynski R. , Current and Future Aspects of Several Adjunctive Treatment Strategies in Polycystic Ovary Syndrome, Reproductive Biology. (2019) 19, no. 4, 309–315, 10.1016/j.repbio.2019.09.006. 31606349

[14] Ke M. , Kang L. , Wang L. et al., CAR-T Therapy Alters Synthesis of Platelet-Activating Factor in Multiple Myeloma Patients, Journal of Hematology & Oncology. (2021) 14, no. 1, 10.1186/s13045-021-01101-6. PMC8191024 34108020

[15] Li J. , Li L. , Liu R. et al., Integrative Lipidomic Features Identify Plasma Lipid Signatures in Chronic Urticaria, Frontiers in Immunology. (2022) 13, 10.3389/fimmu.2022.933312. PMC9370552 35967440

[16] Zhou H. , Nong Y. , Zhu Y. et al., Serum Untargeted Lipidomics by UHPLC-ESI-HRMS Aids the Biomarker Discovery of Colorectal Adenoma, BMC Cancer. (2022) 22, no. 1, 10.1186/s12885-022-09427-1. PMC8943952 35331175

[17] ChenH Z. J. , Zhou H. , Zhu Y. , Liang Y. , Zhu P. , and Zhang Q. , UHPLC-HRMS-Based Serum Lipisdomics Reveals Novel Biomarkers to Assist in the Discrimination Between Colorectal Adenoma and Cancer, Frontiers in Oncology. (2022) 12, 10.3389/fonc.2022.934145. PMC9366052 35965551

[18] Genazzani A. D. , Santagni S. , Rattighieri E. et al., Modulatory Role of D-Chiro-Inositol (DCI) on LH and Insulin Secretion in Obese PCOS Patients, Gynecological Endocrinology. (2014) 30, no. 6, 438–443, 10.3109/09513590.2014.897321. 24601829

[19] Genazzani A. D. , Inositol as Putative Integrative Treatment for PCOS, Reproductive BioMedicine Online. (December 2016) 33, no. 6, 770–780, 10.1016/j.rbmo.2016.08.024. 27717596

[20] Dewailly D. , Robin G. , Peigne M. , Decanter C. , Pigny P. , and Catteau-Jonard S. , Interactions Between Androgens, FSH, Anti-Müllerian Hormone and Estradiol During Folliculogenesis in the Human Normal and Polycystic Ovary, Human Reproduction Update. (2016) 22, no. 6, 709–724, 10.1093/humupd/dmw027. 27566840

[21] Mendoza N. , Diaz-Ropero M. P. , Aragon M. et al., Comparison of the Effect of Two Combinations of Myo-Inositol and D-Chiro-Inositol in Women With Polycystic Ovary Syndrome Undergoing ICSI: A Randomized Controlled Trial, Gynecological Endocrinology. (2019) 35, no. 8, 695–700, 10.1080/09513590.2019.1576620. 30880505

[22] Patel S. , Polycystic Ovary Syndrome (PCOS), an Inflammatory, Systemic, Lifestyle Endocrinopathy, Journal of Steroid Biochemistry and Molecular Biology. (2018) 182, 27–36, 10.1016/j.jsbmb.2018.04.008. 29678491

[23] Wang K. , Li Y. , and Chen Y. , Androgen Excess: A Hallmark of Polycystic Ovary Syndrome, Frontiers in Endocrinology. (2023) 13, no. 14, 10.3389/fendo.2023.1273542. PMC10751361 38152131

[24] Sacchi S. , Marinaro F. , Tondelli D. et al., Modulation of Gonadotrophin Induced Steroidogenic Enzymes in Granulosa Cells by D-Chiro-Inositol, Reproductive Biology and Endocrinology. (2016) 14, no. 1, 10.1186/s12958-016-0189-2. PMC5006365 27582109

[25] Diamanti-Kandarakis E. , Polycystic Ovarian Syndrome: Pathophysiology, Molecular Aspects and Clinical Implications, Expert Reviews in Molecular Medicine. (2008) 10, 10.1017/s1462399408000598. 18230193

[26] Dong F. , Deng D. , Chen H. et al., Serum Metabolomics Study of Polycystic Ovary Syndrome Based on UPLC-QTOF-MS Coupled With a Pattern Recognition Approach, Analytical and Bioanalytical Chemistry. (2015) 407, no. 16, 4683–4695, 10.1007/s00216-015-8670-x. 25860656

[27] Atiomo W. and Daykin C. , Metabolomic Biomarkers in Women With Polycystic Ovary Syndrome: A Pilot Study, Molecular Human Reproduction. (2012) 18, no. 11, 546–553, 10.1093/molehr/gas029. 22809877

[28] Nestler J. E. and Unfer V. , Reflections on Inositol(S) for PCOS Therapy: Steps Toward Success, Gynecological Endocrinology. (2015) 31, no. 7, 501–505, 10.3109/09513590.2015.1054802. 26177098

[29] Wojciechowska A. , Osowski A. , Jóźwik M. , Górecki R. , Rynkiewicz A. , and Wojtkiewicz J. , Inositols′ Importance in the Improvement of the Endocrine-Metabolic Profile in PCOS, International Journal of Molecular Sciences. (2019) 20, no. 22, 10.3390/ijms20225787. PMC6888190 31752081

[30] Li W. , Liu C. , Yang Q. , Zhou Y. , Liu M. , and Shan H. , Oxidative Stress and Antioxidant Imbalance in Ovulation Disorder in Patients With Polycystic Ovary Syndrome, Frontiers in Nutrition. (2022) 9, 10.3389/fnut.2022.1018674. PMC9650267 36386912

[31] Chełchowska M. , Jurczewska J. , Gajewska J. et al., Antioxidant Defense Expressed as Glutathione Status and Keap1-Nrf2 System Action in Relation to Anthropometric Parameters and Body Composition in Young Women With Polycystic Ovary Syndrome, Antioxidants. (2023) 12, no. 3, 10.3390/antiox12030730. PMC10045817 36978978

[32] Camp O. G. , Moussa D. N. , Hsu R. , Awonuga A. O. , and Abu-Soud H. M. , The Interplay Between Oxidative Stress, Zinc, and Metabolic Dysfunction in Polycystic Ovarian Syndrome, Molecular and Cellular Biochemistry. (2025) 480, no. 4, 2015–2023, 10.1007/s11010-024-05113-x. 39266804

[33] Paczkowska K. , Rachoń D. , Berg A. et al., Alteration of Branched-Chain and Aromatic Amino Acid Profile as a Novel Approach in Studying Polycystic Ovary Syndrome Pathogenesis, Nutrients. (2023) 15, no. 19, 10.3390/nu15194153. PMC10574162 37836437

